# Addressing the Black Box of AI—A Model and Research Agenda on the Co-constitution of Aging and Artificial Intelligence

**DOI:** 10.1093/geront/gnae039

**Published:** 2024-05-03

**Authors:** Vera Gallistl, Muneeb Ul Lateef Banday, Clara Berridge, Alisa Grigorovich, Juliane Jarke, Ittay Mannheim, Barbara Marshall, Wendy Martin, Tiago Moreira, Catharina Margaretha Van Leersum, Alexander Peine

**Affiliations:** Division Gerontology and Health Research, Karl Landsteiner University of Health Sciences, Krems, Austria; Interdisciplinary Center for Gender Studies, Switzerland and Goa Institute for Management, University of Bern, Goa, India; School of Social Work, University of Washington, Seattle, Washington, USA; Recreation and Leisure Studies Department, Brock University, St. Catharines, Ontario, Canada; University of Graz, Graz, Austria; Department of Communication Studies, Ben-Gurion University of the Negev, Beer-Sheva, Israel; Department of Sociology, Trent University, Peterborough, Ontario, Canada; Department of Health Sciences, Brunel University London, Uxbridge, Middlesex, UK; Department of Sociology, Durham University, Durham, UK; Department of Digital Culture, Innovation and Communication, Faculty of Humanities, Open University of the Netherlands, Heerlen, Limburg, The Netherlands; Department of Digital Culture, Innovation and Communication, Faculty of Humanities, Open University of the Netherlands, Heerlen, Limburg, The Netherlands

**Keywords:** Ageism, Algorithm, Datafication, Gerontechnology

## Abstract

Algorithmic technologies and (large) data infrastructures, often referred to as Artificial Intelligence (AI), have received increasing attention from gerontological research in the last decade. Although there is much literature that dissects and explores the development, application, and evaluation of AI relevant to gerontology, this study makes a novel contribution by critically engaging with the theorizing in this growing field of research. We observe that gerontology’s engagement with AI is shaped by an interventionist logic that situates AI as a black box for gerontological research. We demonstrate how this black box logic has neglected many aspects of AI as a research topic for gerontology and discuss three classical concepts in gerontology to show how they can be used to open various black boxes of aging and AI in the areas: (a) the datafication of aging, (b) the political economy of AI and aging, and (c) everyday engagements and embodiments of AI in later life. In the final chapter, we propose a model of the co-constitution of aging and AI that makes theoretical propositions to study the relational terrain between aging and AI and hence aims to open the black box of AI in gerontology beyond interventionist logic.

Artificial intelligence (AI) and algorithmic technologies have gained increasing relevance and attention in society, including its health and care systems (Berridge & Grigorovich, 2022; Lukkien et al., 2023). Although it is difficult if not impossible to give a clear definition of AI, the term usually refers to some form of algorithmic automation—for instance, automated decisions, decision support, or classifications—based on large quantities of data about behaviors of people.

For instance, social media, streaming services or fitness trackers use large amounts of data to make recommendations about what news items to consume, what movies to watch or how much calories still need to be burned in a day. Companies and government organizations increasingly rely on automated decision making, fed by data about a client’s or citizen’s identity and behaviors, for instance in granting mobile phone contracts, deciding about access to social benefits schemes, or policing traffic offenders. Large Language Models (LLMs) such as ChatGPT use sophisticated autocomplete algorithms, operating on large quantities of previously published language to generate texts that appear meaningful.

It is increasingly clear that AI also plays a role in the lives of older people. For one, the everyday lives of older people are no exception to the prevalence of data-collecting and -processing technologies like smartphones, connected cars, or smart speakers. At the same time, much hope is placed on AI-based technologies specifically designed for older people, for instance in the form of decision support systems in dementia care, remote risk detection, and monitoring systems to detect falls or deviations from “normal” daily routines, or social robots that can hold conversations to mitigate loneliness. Much attention and large-scale financial investments are directed to such AI in gerontechnology (Rubeis, 2020). The overriding hope is that AI in gerontechnology will support health and care professionals (e.g., in clinical decision making, remote monitoring, or predictive analysis), and ultimately enable older adults to live and age autonomously (Chen, 2020).

The relevance of AI for the lives of older people has been recognized in gerontology (Chu et al., 2022; Lukkien et al., 2023). So far, however, available studies have focused on evaluating the “impact” of AI technologies on the lives of older people, predominantly in the context of formal and informal care (Loveys et al., 2022). Critical engagement with theory has been largely absent in these studies, which are typically designed as intervention studies—a specific AI technology is implemented temporarily in the lives or care environments of a selected group of older people to measure how far certain parameters such as depression symptoms, quality of life or agitation changed (Loveys et al., 2022).

In this study, we discuss the need for gerontology to more deeply engage with theorizing in this growing field of AI and aging research. Our main argument is that gerontology’s focus on intervention studies goes hand in hand with what Peine and Neven (2019) have called “interventionist logic.” This logic analytically separates the lives of older people from the design and use of technologies so that technology can neatly be conceptualized as an intervention with defined and measurable parameters for its success (or failure). It thus renders invisible and makes inaccessible to critical theoretical reflection, the dynamics of aging and technology relations in both the worlds of technological design and the lives of older people.

Hence, the interventionist logic renders AI as a black box (Latour, 2000) in gerontology, making invisible the construction of aging in AI design, its embedding in socio-material infrastructures, and the engagements of older adults with it. To rectify this, we draw on the co-constitution of aging and technology (Peine & Neven, 2021) which studies how aging and technology come into existence in relation to each other. This notion allows us to conceptually explore the relationship between aging and AI, highlight the sociomaterial associations that currently exist between aging and AI, and outline questions for future gerontological research on aging and AI. This study, therefore, is a timely intervention that seeks to broaden gerontology’s perspective on AI and open up this topic for gerontological debate and research beyond interventionist logic.

It is a core argument of our approach that the term AI is vague and fluid because it is used in different ways by different parties, often in strategic and deliberately obfuscating ways. For us, it is thus an empirical question of what AI is and does in different contexts, and we outline a research agenda that aims to engage more deeply with the question of what AI is in the lives of older people towards the end of this article. We try to be specific, though, about the core technologies and processes that the products and services have in common that populate discourses around AI: Big data infrastructures and algorithmic decision making and classification systems. Broussard (2018) provides a succinct and easy-to-understand introduction to the technological core of many AI technologies. Our description of AI as automation based on large quantities of data has also been inspired by the work Emily Bender (2023).

## Black-Boxing AI in Gerontology

The figure of the black box is often used to highlight forms of opaqueness in the design and deployment of AI systems (Jarke & Heuer, 2024). On a technical level, this opaqueness stems from algorithmic processes that infer correlations, representations, and categories based on large amounts of data. The ways in which these outputs are created often remain incomprehensible to human beings (Carabantes, 2020), partly because companies refuse transparent data documentation practices. The figure of the black box is also used to describe how AI is mystified (Søraa, 2023) as an entity that detects patterns beyond human intelligence or imagination (Campolo & Crawford, 2020). Such mystification can be seen as a deliberate attempt to distract from the more immediate challenges that arise from the marketization of AI in an age of surveillance capitalism (Zuboff, 2019). Black-boxing also relates to the way in which AI systems are embedded in health care and long-term care infrastructures and the lives of its users. These embeddings and the associated changes in decision-making processes are widespread, yet they often remain opaque to those interacting within them.

Hence, the black-boxing of AI not only exists due to technological complexity, but it may also be intentionally or unintentionally made to be so by a variety of actors. These actors, we argue, include gerontologists who neglect technological development, implementation, and related social processes as objects of scientific study. We hence argue that a black box can also be understood as an empirical and conceptual problem that can and should be “opened” or “unpacked” (Bucher, 2018) by those who study aging and technology.

In what follows, we aim to open the black box of AI in gerontology by revisiting a range of classical concepts in gerontology and discussing how they can be used to open various black boxes of aging and AI. Opening these black boxes, we argue, holds two potentials for gerontological research: On the one hand, it offers possibilities for more in-depth engagements with AI as a gerontological topic of research. On the other hand, it also offers new ways of theorizing the relationship between aging and AI that go beyond interventionist logic and instead, focus on the co-constitution of aging and technology (Peine & Neven, 2019, 2021). We conclude by offering a model of the co-constitution of aging and AI that makes theoretical propositions to study the relational terrain between aging and AI beyond interventionist gaze.

### The Datafication of Aging

In Disciplining Old Age, Katz (1996) employed a Foucauldian analysis of the classification practices that constitute aging bodies as inherently senescent and risky, yet also amenable to discipline and remediation. He provided a portrait of aging bodies as historically configured through webs of knowledge and power. Such classification practices are at the heart of AI and algorithmic technologies (Joyce et al., 2021), which makes the webs of knowledge and power that constitute old age today increasingly technologically mediated, more durable and persistent.

One way of unpacking the black box of AI in the context of aging lies in exploring how AI is not only a technological system, but a web of knowledge that constitutes aging bodies as measurable and quantifiable. AI systems tend to prioritize information that can be quantified, categorized, and classified. Based on these data, AI systems produce output that is taken to be objective, authoritative, and fair. This suggests the collection of data on older populations is universally able to represent an objective, external reality about aging and views the application of technological knowledge as the most effective and economical approach to solving the “problems” linked to population aging (Moreira, 2017). This techno-solutionist thinking, however, tends to black box the situated nature of both algorithms and data, as it makes the role of humans in making and constructing these data, and the “constructed, polyvalent nature of human data” (Joyce et al., 2021) invisible.

Another way of unpacking the black box of AI in gerontology hence lies in theorizing data beyond such a techno-solutionist logic. For example, Beer (2019) refers to the value offered by data analytics as comprising a “data imaginary,” promising speed, accessibility, insight, prediction, and efficiency. When data imaginaries are enacted, for example, through ambient monitoring technologies for aging in place, AI-driven analytics are promoted as the most cost-efficient and “smart” solutions to caring for an aging population. However, as Beer (2016), p. 60 remarks, such forms of measurement are not only powerful for what they capture but also for what they conceal or ignore. This truncated data imaginary risks devaluing or obscuring other kinds of knowledge or experience, rendering factors outside of the machine’s calculations as less relevant to the production of knowledge. Through datafied classification and categorization, the visibility of an older body is limited to the data produced by AI systems, which is then “aggregated and itemized into risk assessments and patterns of behavior” (Ellison et al., 2022). Older adults get placed into categories with consequences for their lives—they can become “fallers,” “at risk,” “frail,” “aggressive,” in need of residential care or “untrustworthy” reporters (Berridge & Grigorovich, 2022), while their subjective experiences of these categories become less relevant forms of knowledge.

### The Political Economy of Aging and AI

Political economy perspectives in gerontology (Estes et al., 1982) have articulated the ways in which capitalism and neoliberal profit imperatives drive interest in older adults, ultimately framing them as “profit-making commodities” (Estes, 1993). There is a similar profit-making logic involved in massive investments of policymakers and companies in the development of AI gerontechnology (Sadowski, 2019). As the black box logic of AI tends to make commodification and profit generation through AI invisible, one way of unpacking the black box lies in engaging more deeply with the commodification of aging in the political economy of AI and asking which forms of profit generation become relevant as AI for older adults is developed and implemented.

In the political economy of aging and AI, older adults tend to be viewed as data suppliers who generate profits for AI companies (Birch & Cochrane, 2022). Chu et al. (2022) note that there is hardly enough data about older adults available to train AI models toward the needs of this population. Available data infrastructures often show explicit or implicit age-related bias (Fernández-Ardèvol & Grenier, 2022). This points to a major structural problem for the creation of inclusive and fair AI systems (Stypinska, 2022 ) but also enables AI companies to collect “unique aged-data” to create new market segments. However, even if the data are derived from, for example, racially diverse or economically marginalized older populations, any divergence gets subsumed within the neoliberal logic of difference (Ludwig, 2016; McNay, 2009) rather than the politics of intersectionality (Ranjan-Rankin, 2018). In this way, efforts to create more representative data sets of older adults commodify “difference” as marginalized groups of older adults become unique and valuable data suppliers. For AI companies, inclusion efforts thus become a new means of generating profits through the “differential quality of user data and engagement” (Birch & Cochrane, 2022, p. 51).

Furthermore, a political economy perspective on AI gerontechnologies enables questioning the role of (paid and unpaid) labor which is necessary to implement AI systems. Even though AI is often perceived as a neutral artifact that “parachutes into” the lives of older adults, specific uses of AI require contextualization and local embedding (Lukkien et al., 2023). Consequently, recent research has stressed the relevance of invisible—and at times unpaid—caregiving, which is key to achieving the implementation of AI in diverse contexts of aging (Gallistl & von Laufenberg, 2023). This not only includes the work of software designers and programmers but also work of health care professionals, care staff in long-term care organizations, or the data work that older adults and family carers need to provide to make AI systems run in practice.

A political economy perspective on aging and AI demands reflection about and analysis of the “ground-truthing, programming and formulating” (Jaton, 2021) that happens in AI companies and transparency about what data a company is collecting with what purpose and how profit is generated through this data (Tucker, 2022).

### Everyday Embodiments of AI in Later Life

Cultural gerontology has put forward an embodied understanding of old age and later life (Twigg & Martin, 2015). This has sought to move away from a bio-medical gaze on the older body (e.g., Öberg, 1996) and instead focuses on how aging is embodied as an everyday experience. The embodiments and engagements with AI in the everyday lives of older adults are often overlooked and older adults’ agency is rarely discussed in the context of AI (Neves et al., 2023). However, these engagements are crucial to enhance the design and implementation of technologies into the lives of older adults (van Leersum et al., 2023). One way of unboxing the black box of AI in gerontology lies in theorizing the ways in which datafied embodiment (Lupton et al., 2022) gains relevance in the lives of older adults, and in questioning how older adults perceive, make sense of, or stand in opposition to the implementation of AI and its related data practices (for discussion of refusal, see Berridge & Grigorovich, 2022, 2023; Brewer, 2022).

Understanding older adults’ embodiments and agency in the context of AI includes further unpacking the ways in which the everyday lives of older people are becoming sites of datafication, monitoring, and surveillance (Dalmer et al., 2022; Ellison et al., 2022). This might also include making visible how the embodiment of aging in everyday life is more-than-human, and how diverse materialities (including AI systems) are part of the constitution of aging in the lives of older adults. Traditionally, gerontology has theorized aging as a human phenomenon, that happens to or within an aging body. These boundaries of aging are, however, increasingly blurred as datafied forms of embodiment and data doubles become part of the everyday experience of aging. Another way of unpacking the black box of AI in gerontology lies in exploring how diverse forms of materiality—including sensors, wearables, social or assistive robots, or other monitoring devices—become an integral part of the experience of aging. These entanglements between humans and non-humans can be observed and mapped as a means to understand more about aging as a more-than-human phenomenon (Gallistl & Wanka, 2023, Wanka & Gallistl, 2018).

The focus on everyday embodiments and engagements of AI also offers a novel perspective on ethics and values that underpin AI. So far, ethical debates on AI in aging research have largely been informed by a principlist approach, which has narrowed ethical reflections to balancing risks and benefits of a certain AI system (Grigorovich & Kontos, 2020). An everyday perspective on ethics in AI highlights that ethical questions cannot be captured and solved at one point in time but require ongoing attention to the multiple values that inform the development, implementation, and use of AI (van Hees et al. 2023, Gallistl & Wanka, 2022). Such a valuation approach toward ethics in AI might not seek to define and solve ethical questions but rather explore the practices of valuation through which manifold forms of value are “produced, diffused, assessed, and institutionalized” (Lamont, 2012: 201) throughout the lifecycle of an AI system. It might ask, for example: What are diverse notions of “good” that inform the development and the implementation of an AI system? How are they re-constituted in such development and implementation?

## Conclusion: Co-constitution of Aging and Artificial Intelligence—A Research Agenda and Model

**Figure 1.**
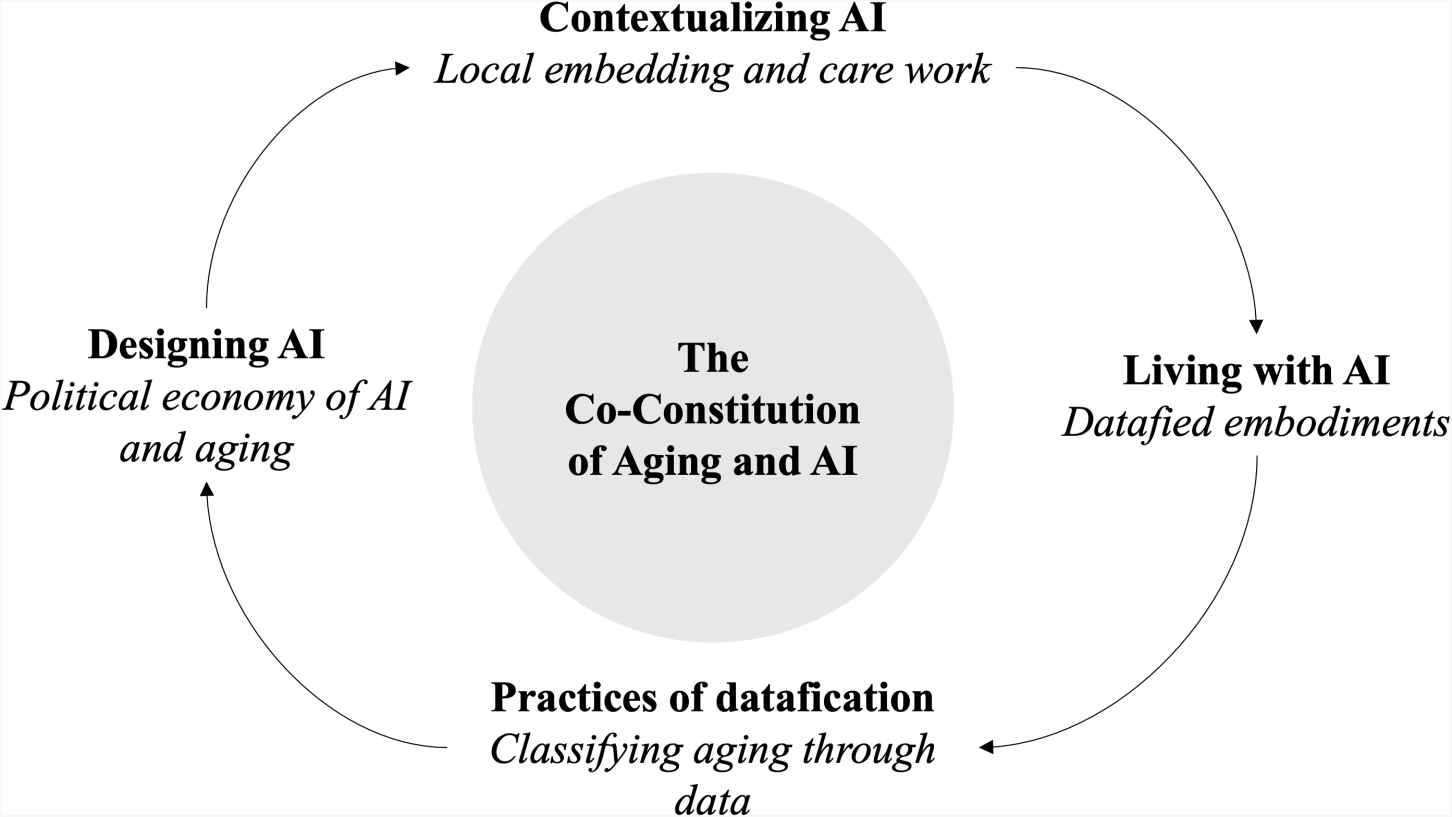
Model of the co-constitution of aging and AI (developed further from Peine & Neven, 2021).

Considerable public investment in AI, particularly in the care for older adults, means that relevance of AI for older adults will grow, and so will the need to study AI as a gerontological subject matter. We conclude by putting forward four pathways for research on the relational terrain between aging and AI (Figure 1). These pathways build on the overarching conceptual themes we have drawn out from prior gerontological scholarship: classification in knowledge and power, political economy, and embodiment. This model proposes four arenas in which the co-constitution of aging and AI can be studied.

First, gerontology ought to interrogate practices of designing AI and its related logic of commodification and value creation within a political economy of aging. We propose that gerontological research needs to engage with the ways in which AI is imagined, developed, and evaluated for various older target groups, and critically analyze the images of aging that guide this design. This might also include asking which myths are created around AI for older adults with the aim of creating hype and investment about these systems (Hoffman et al., 2022) and exploring the ways in which AI is marketed across various target groups (e.g., health care systems, the long-term care industry, older adults, family care partners, etc.).

It also means engaging critically with persisting and emerging forms of exploitation within a political economy of AI and aging. As a field that is “data-rich” (Bengtson & Birren, 1988) gerontology is well-positioned to investigate the representation of older adults in data infrastructures that are available to train AI, as well as the consequences of under- or over-representation in these infrastructures. Examining potential harms is an ethical imperative and will be most impactful when it is responsive to the realities of those who experience marginalization, including 2SLGBTQI+ (two-spirit lesbian, gay, bisexual, transgender, queer, intersex, + to include people who identify as part of sexual and gender diverse communities who use additional terminologies), BIPOC (Black, Indigenous and People of Color), disabled, and economically vulnerable older people and care workers (Berridge & Grigorovich, 2022).

Second, we invite gerontologists to explore how AI systems for older adults are practically contextualized, implemented, and locally embedded in care arrangements and everyday life activities; and ask how these care arrangements are changing through the embedding of AI and big data logics. Recent reviews have suggested that there is a lack of knowledge on how innovation through AI is practically achieved in context (Lukkien et al., 2023). On the one hand, this is because AI companies aim to offer somewhat standardized and scalable solutions, but it is also because AI is portrayed as an objective and neutral technological actor, which tends to make the human labor involved in the creation and implementation of these systems invisible (Gallistl & von Laufenberg, 2023).

Third, our work has highlighted the need to explore empirically how older adults make sense of, engage and tinker with AI in their everyday lives. Discourses that portray older adults as incompetent, uninterested, or invisible users of technologies (Mannheim et al., 2022) tend to black-box the active engagements of older adults with technologies. However, research in the field of Socio-gerontechnology (Peine et al., 2021) has highlighted that older adults—including people with high care needs—routinely and actively engage with technological innovations (Gallistl et al., 2021), underscoring the importance of older adult’s agentic engagement and subjectivity in research on AI. Recent work on explainability of AI has proposed to focus on sense-making practices as a crucial element in understanding how people perceive and understand AI (Papagni et al., 2023). There is significant room in the context of aging for work on explainability, as well as expanding participation in AI development and governance.

Fourth, gerontology ought to interrogate the meanings of data in the context of AI and the practices of datafication that go hand in hand with the development and implementation of AI. Many AI systems rely on massive amounts of data available through the health and care sectors with the built-in assumption that these data are neutral and truthful representations of reality (Hoffman et al., 2022). In contrast, we highlight that what we understand as data, and the value we connect to it in the context of aging, is contingent, ambivalent, and ever-changing. We, therefore, invite gerontologists to engage with data imaginaries (Beer, 2019), to question gerontology’s values and expectations around data, and to explore the research culture that forms around these values. This also means exploring the ways in which data are collected, curated, and used to build AI for an aging society, as well as questioning what kinds of reality about aging are represented in and created through these data.

Finally, we highlight the need to reflect on how the relationship between aging and AI might be imagined otherwise. The emergence of AI applications poses several ethical questions and challenges, but it also presents an opportunity to ask how socio-technical arrangements can bring about better futures (Joyce et al., 2021). This holds true for imagining a more age-inclusive and age-friendly society. As Onuaha articulated a recent interview (2022), technology is created in the service of something, and when we do not have clarity about what aims it is in service of, “it will just default to supporting the dominant model of power that exists at the time, or the means to which you can attain power.” We invite gerontology to ask, what is the purpose or benefit of AI applications in gerontechnology beyond the service of capitalist ends? And what are the liberatory purposes that we may reorient toward instead? For example, can we develop AI gerontechnolgies to prevent the transformation of aging subjects into data capital to be exchanged or monopolized by AI companies for profits? Can we disentangle efficiency from care quality in a way that is accountable to older adults? Can AI applications support various ways of aging without or beyond commodification, profit generation, or cost reduction?

Drawing on empirical engagements with the four areas of practice we outlined in our model, we hope that gerontologists will explore these questions as part of a gerontological research agenda and further develop the theoretical tools needed to imagine socio-technical futures of aging that are grounded in the contexts of peoples’ lives rather than today’s context of AI hype.

## Data Availability

The authors do not report data and therefore the pre-registration and data availability requirements are not applicable.
